# A validated model for early prediction of group A streptococcal aetiology in necrotising soft tissue infections using minimal patient data

**DOI:** 10.1186/s12916-025-04593-y

**Published:** 2026-01-10

**Authors:** Sonja Katz, Jaco Suijker, Steinar Skrede, Annebeth Meij-de Vries, Anouk Pijpe, Anna Norrby-Teglund, Laura M. Palma Medina, Jan K. Damås, Ole Hyldegaard, Erik Solligård, Mattias Svensson, PerAID/PerMIT/INFECT study group, Steinar Skrede, Anna Norrby-Teglund, Ole Hyldegaard, Mattias Svensson, Michael Nekludov, Ylva Karlsson, Per Arnell, Muhammad Afzal, Helena Bergsten, Lydia Bosnak, Bavya Chakrakodi, Puran Chen, Johanna Emgård, Linda Johansson, Julius Juarez, Srikanth Mairpady Shambat, Nikola Siemens, Johanna Snäll, Julia Uhlman, Takeaki Wajima, Martin B. Madsen, Daniel Bidstrup, Nina F. Bærnthsen, Julie V. Clausen, Anna Damgaard, Gladis H. Frendø, Martin Forchammer, Marco Hansen, Morten F. F. Hedetoft, Karen L. Hilsted, Diana Isaksen, Erik C. Jansen, Josefine Kofoed, Anette Lilja, Lærke B. Madsen, Rasmus Müller, Isabel S. Nielsen, Emilie M. J. Pedersen, Marie W. Petersen, Anders Perner, Peter V. Polzik, Frederikke Ravn, Folke Lind, Anders Kjellberg, Erik von Oelreich, Peter Kronlund, Sverre Kullberg, Ola Friman, Lisa Hellgren, Anna Granström, Anna Schenning, Sandra Carlsson, Trond Bruun, Torbjørn Nedrebø, Oddvar Oppegaard, Eivind Rath, Marianne Søvik, Hanne Søyland, Dag Benoni, Hans Lycke, Joakim Trogen, Kerstin Ohlauson, Dietmar H. Pieper, Singh Chhatwal, Andreas Itzek, Anshu Babbar, Robert Thänert, Jörn Hoßmann, Eva Medina, Domenica Hamisch, Israel Barrantes, Patric Nitsche-Schmitz, Astrid Dröge, Katja Mummenbrauer, Francois Vandenesh, Sylvere Bastien, Jessica Baude, Anne Tristan, Erno Lindfors, Francois Bergey, Christoph Reschreiter, Bernhard Ronacher, Matthias Pilecky, Eytan Ruppin, Matthew Oberhardt, Raphy Zarecky, Malak Kotb, Karthickeyan Chellakrishnan, Santhosh Mukundan, Suba Nokala, Doreen Marsden, Kristoffer Strålin, P. P. M. van Zuijlen, Marco Anteghini, Vitor A. P. Martins dos Santos, Edoardo Saccenti, Knut Anders Mosevoll, Vitor A. P. Martins dos Santos, Edoardo Saccenti

**Affiliations:** 1 Laboratory of Systems and Synthetic Biology, Wageningen University and Research, Wageningen, The Netherlands; 2 Department of Radiology and Nuclear Medicine, Erasmus MC, Rotterdam, The Netherlands; 3 Alliance of Dutch Burn Care, Red Cross Hospital, Vondellaan 13, Beverwijk, 1942 LE The Netherlands; 4 Amsterdam UMC Location Vrije Universiteit Amsterdam, Plastic, Reconstructive and Hand Surgery, De Boelelaan 1117, Amsterdam, The Netherlands; 5 Amsterdam Movement Sciences, Tissue Function and Regeneration, Amsterdam, The Netherlands; 6 Department of Clinical Science, University of Bergen, Pb 7804, Bergen, 5020 Norway; 7 Department of Medicine, Haukeland University Hospital, Pb 1400, Bergen, 5021 Norway; 8 Pediatric Surgical Centre, Emma Children’s Hospital, Amsterdam UMC, Amsterdam, the Netherlands; 9 Center for Infectious Medicine, Karolinska Institutet, Karolinska University Hospital, Huddinge, Sweden; 10 Norwegian University of Science and Technology, Trondheim, Norway; 11 Department of Infectious Diseases, St. Olav’s Hospital, Trondheim University Hospital, Trondheim, Norway; 12 Centre of Molecular Inflammation Research, Department of Clinical and Molecular Medicine, Norwegian University of Science and Technology, Trondheim, Norway; 13 Department of Clinical Medicine, University of Copenhagen, Copenhagen, Denmark; 14 Department of Anaesthesia, Centre of Head and Orthopaedics, Copenhagen University Hospital, Copenhagen, Denmark; 15 Department of Research, Innovation, Education and Health Service Development, Møre og Romsdal Hospital Trust, Ålesund, Norway; 16 Department of Bioprocess Engineering, Wageningen University & Research, Wageningen WE, The Netherlands; 17 LifeGlimmer GmbH, Berlin, Germany

**Keywords:** Amputation, Flesh-eating bacteria, Machine learning models, Predictive modelling, *Streptococcus pyogenes*, Septic shock, ICU stay

## Abstract

**Background:**

Necrotising soft tissue infections (NSTI) are life-threatening conditions caused by diverse bacteria. Treatment strategies have remained largely universal and unchanged, and only modest improvements in patient outcomes have been observed. Emerging insights into NSTI pathogenesis may enable more targeted approaches. Because microbial aetiology is central to guiding appropriate therapy, we aimed to develop and externally validate machine learning models capable of predicting microbial aetiology using only data available at an early stage. In parallel, we explored whether similar models could predict selected clinical endpoints related to surgical management, patient handling, and organ support.

**Methods:**

We used data from the INFECT study, an international multicentre prospective cohort investigating NSTI characteristics and pathogenesis. A total of 409 adults with surgically confirmed NSTI were enrolled between February 2013 and June 2017 from five Scandinavian hospitals. More than 700 clinical variables were collected from hospital admission to intensive care unit entry. Machine learning models were developed to predict the presence of *Streptococcus pyogenes* (GAS, Group A *streptococcus*) and five clinical endpoints: risk of amputation, size of skin defect, maximum skin defect size, length of intensive care (ICU) stay, and need for renal replacement therapy. Unsupervised variable selection was implemented, and Shapley Additive explanations were used for model interpretability. External validation employed a retrospective multicentre cohort of 216 NSTI patients treated in 11 Dutch hospitals between January 2013 and December 2017.

**Results:**

Eight presurgical variables (age, diabetes, affected area, prior surgical intervention, and blood creatinine and haemoglobin concentrations) were sufficient for predicting GAS aetiology with high discriminatory power. Performance was good in both the development cohort (ROC-AUC 0.828; 95% CI 0.763–0.883) and the external validation cohort (ROC-AUC 0.758; 95% CI 0.696–0.821). Prediction of clinical endpoints related to surgical management, ICU stay, and organ support was unsuccessful.

**Conclusions:**

We developed and externally validated a model predicting GAS aetiology in NSTI using presurgical data alone. Early identification of GAS may improve clinical handling and support tailored decisions on treatment and infection control, including management of close contacts and reduction of hospital transmission risk.

**Supplementary Information:**

The online version contains supplementary material available at 10.1186/s12916-025-04593-y.

## Background

Necrotising soft tissue infections are rapidly spreading infections of subcutaneous tissue, fat, fasciae, and muscle, associated with septic shock and considerable mortality rates [1]. Pivotal requirements for favourable outcomes are early recognition, prompt surgical removal of infected tissue, appropriate antimicrobials, and frequent surgical revisions. Despite ongoing research, there has been modest improvement in patient outcomes over time [2], and treatment strategies in NSTI have remained unchanged over time and are most commonly universal.

A major challenge in NSTI is to arrive at the diagnosis early and timely. Several preoperative diagnostic score systems to rule in NSTI and rule out cases with cellulitis have been built, based on routinely available blood tests [3, 4], but have been evaluated usually with negative results [5]. Preoperative blood tests can be used for prognostication [6–9]. We have identified thrombomodulin as a candidate biomarker for NSTI [10], and nine biomarkers discerning beta-haemolytic streptococcal NSTI from streptococcal cellulitis [11]. However, tests for the rapid measurement of neither diagnostic nor prognostic markers are available today for clinical use in an emergency setting.

Clinical score systems that show promise to rule in NSTI have been proposed, but they are not widely in use or recommended by current guidelines for NSTI management [11–13].

Other approaches have been proposed to aid NSTI diagnosis, including the use of standard imaging workup, which is discouraged [14]. Fluid analysis of aspirates from the infected area has been presented as a potential rapid diagnostic test [15], but the technique is prone to risks for high interrater variability, making this a possibly unreliable tool. Also, intraoperative measures to establish the NSTI diagnosis have been investigated, including pre-operative histopathology [16], which has later been shown to have low accuracy in suspected NSTI [17].

Many recent advances in the understanding of pathogenesis in NSTI subcategories [18, 19] are still to be exploited, although they may lay the foundation for improvements in targeted clinical handling.

We have previously developed a machine learning-based predictive model to support clinical decision-making for NSTI patients, and we showed that such a model can accurately predict 30-day mortality [20] using only sixteen clinical parameters/variables collected within the initial 24 h of ICU admission. The model attained a prediction of 30-day mortality more accurate than commonly used clinical scoring systems, like the Simplified Acute Physiology Score II (SAPS II) [21] and the Sequential Organ Failure Assessment (SOFA) score [22], used to predict mortality in ICU patients.

In this study we aimed to explore whether it is possible to establish a rapid substantiation of Group A *Streptococcus* aetiology in NSTI cases using patient data, collected at different stages, from hospital admission to admission to intensive care unit. Of particular interest was the possibility of using data collected before surgery as such an early substantiation of GAS aetiology can affect and impact positively early clinical decisions.

To the best of our knowledge, a simple score to substantiate GAS aetiology in NSTI has to date not been proposed. We suggest that clinical evaluation to establish a likely GAS aetiology in NSTI requires extensive clinical experience, which is rare. Here we offer a tool to guide also less experienced colleagues. It is well documented that undertreatment in NSTI is frequent.

Timely knowledge about GAS aetiology can help navigate the difficulties and pitfalls characteristics that are relevant for the treatment and management of GAS NSTI: these include microbial resistance towards clindamycin [23], production of biofilm in soft tissue [24, 25], as well as in cases where a possible administration of IVIG is considered [26].

Unlike polymicrobial NSTI, in GAS NSTI there is a generalised need for early selection of hospital hygiene measures, including use of patient isolate rooms following surgery, to lower the risk for hospital spread [27]. Debate is also ongoing on whether to treat household members of patients with invasive GAS infection (iGAS) [27]. For this treatment to be effective, it should be initiated early [28].

Contextually to the development of a computational model for the GAS aetiology we explored the possibility of using the same patient data to predict several clinical endpoints of relevance in the NSTI context: surgical aspects (risk of amputation, size of skin defect after fist surgery, maximum skin defect size), as well as length of ICU stay, and need for organ support (need for renal replacement therapy). The prediction models were developed and trained using data collected from 409 NSTI patients enrolled within the INFECT study from five Scandinavian hospitals and externally validated on data from a cohort of 211 patients from 11 Dutch centres. Results show that while it is not possible to predict surgery and management endpoints, it is possible to predict the GAS aetiology with relatively high accuracy in both the development and validation cohorts, by using only eight parameters usually collected at early stage (age, diabetes, different anatomical locations of infection, prior surgical intervention, blood creatinine and haemoglobin concentrations).

This proposed model for predicting GAS presence in NSTI patients is intended to be used as a decision-support tool, and a supplement that can increase awareness and guide several of clinicians’ decisions, not restricted to considerations on use of IVIG.

## Methods

### Study design

#### Development cohort

Subjects and data of the patients cohort used to develop and train the prediction models were obtained from the INFECT study (https://permedinfect.com/; registration number NCT01790698, ClinicalTrials.gov), an international, multicentre, prospective cohort study with patients with NSTI included prospectively at five Scandinavian hospitals (Additional file 1: Note 1). A total of 409 patients above the age of 18 and with surgically confirmed NSTI cases were enrolled between February 2013 and June 2017. Extensive demographic and clinical data, treatment results, outcomes and data collection protocols have been previosly published and analyzed [29, 30].

#### Validation cohort

 To be used for validation of predictive models were obtained from a Dutch retrospective multicentre cohort comprised of 216 patients admitted for acute treatment of NSTI to 11 centres (Additional file 1: Note 2) between January 1, 2013, and December 31, 2017, irrespective of a subsequent ICU admission. Patient characteristics have been previously described [31].

#### Clinical endpoints

Six clinically relevant NSTI endpoints were selected through semi-structured interviews as previously described [20] and were used for machine learning modelling. The selected endpoints encompass different clinical aspects of NSTI. Bacterial aetiology: presence of GAS (binary). Surgical aspects: risk of amputation (binary); size of skin defect after first surgery, percentage of body surface (continuous); maximum skin defect size, percentage of body surface (continuous). Patient management: length of ICU stays (continuous). Organ support: need for renal replacement therapy (RRT) (binary). The binary endpoints were coded as 0–1 (absence-presence, no-yes). An overview of the number of patients included and label balance for each clinical endpoint can be found in Additional file 1: Table 1.

**Table 1:** Overview of variables yielded through unsupervised variable selection. Explanations: ^1^ at arrival at specialised hospital; ^2^preoperative (preop): before the first surgery, which is before ICU admission. ^3^Preadmission: upon ICU admission. ^4^Baseline (BL): during the first 24 h in the ICU. ^5^Surgery before: surgery within 4 weeks prior to NSTI. For each time point, records for 45, 56, 723 and 764 variables were available. Entry indicates variables measured at hospital admission. Baseline refers to first 24 h of ICU admission. (see also Fig. 1a) A more detailed variable description can be found in Additional file 1: Table S2

Entry	Pre-surgery	Post-surgery	Baseline
(6/45 variables)	(8/56 variables)	(9/723 variables)	(14/762 variables)
Affected^1^: upper arm	Affected^1^: upper arm	Affected^1^: upper arm	Affected^1^: upper arm
Affected^1^: lower arm	Affected^1^: lower arm	Affected^1^: lower arm	Affected^1^: lower arm
Affected^1^: anogenital area	Affected^1^: anogenital area	Affected^1^: anogenital area	Affected^1^: anogenital area
Surgery before^5^	Surgery before^5^	Surgery before^5^	Surgery before^5^
Diabetes	Diabetes	Diabetes	Diabetes
Age	Creatinine^2^	Creatinine^2^	Creatinine^2^
	Haemoglobin^2^	Haemoglobin^2^	Haemoglobin^2^
	Age	Creatinine^3^	Creatinine^3^
		Anatomical site sampled	Systolic BP (lowest)^4^
			Creatinine^4^
			Noradrenaline^4^
			Platelets^4^
			Lactate^4^
			Glucose^4^

### Statistical and data analysis methods

#### Data preprocessing

**Fig. 1:**
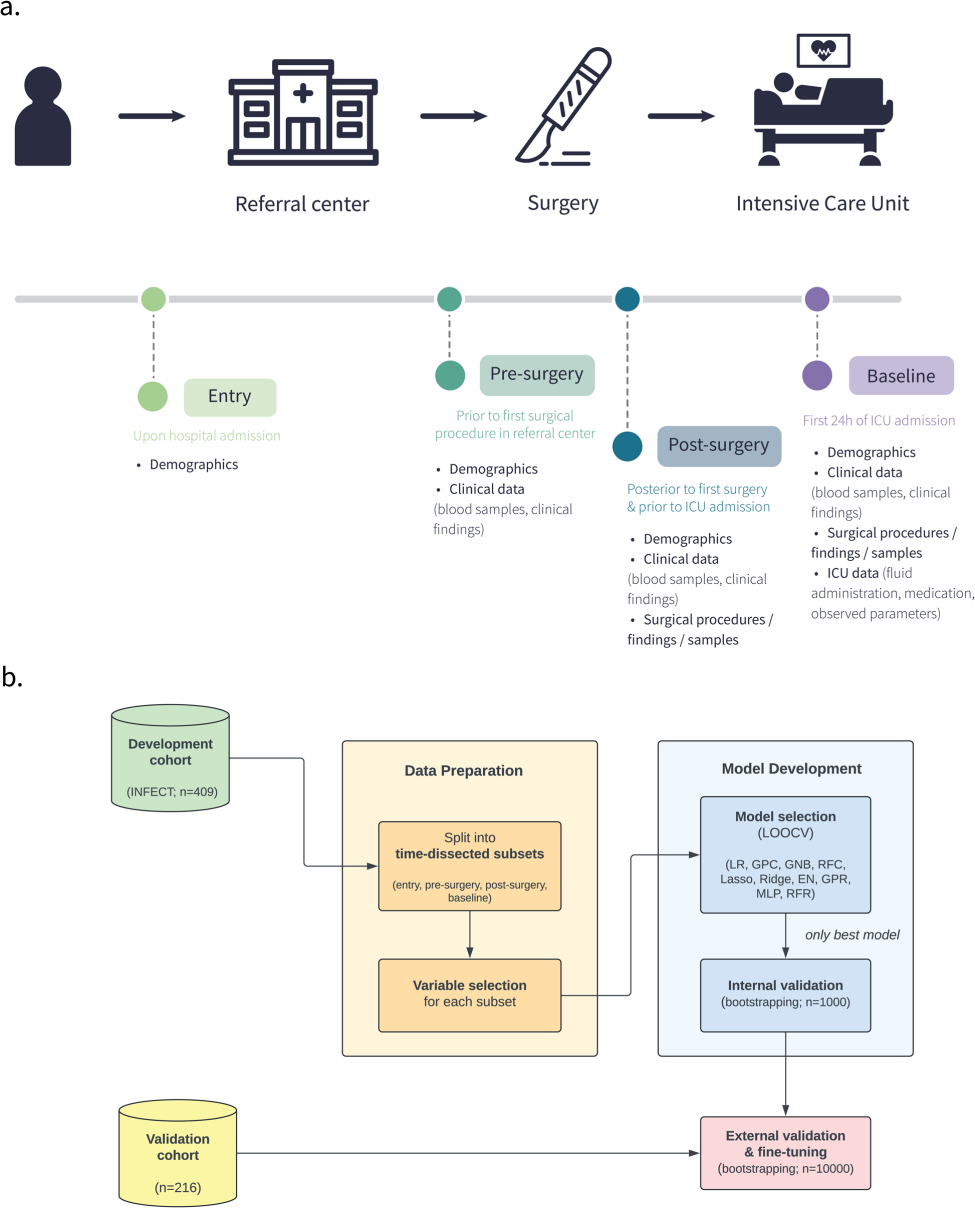
Structured flowcharts detailing the developmental and validation process of the machine learning models. **a** The INFECT study cohort (409 patients) was split into the time-dependent subsets *entry*,* pre-* and *post-surgery* at referral centre, *baseline*, depending at which stage data was available. **b** Developmental and validation process of the machine learning models: the same pipeline was implemented for every clinical outcome assessed. External validation was exclusively pursued concerning the bacterial aetiology (presence of Group A *Streptococcus*). *n*: number of patients/iterations; LOOCV: leave-one-out cross-validation

For the development cohort, data from the INFECT study included over 700 clinical variables, collected from hospital admission to ICU admission [29]. Variables were categorised depending on their availability at four time points: *entry* (upon hospital admission, 45 variables), *pre-surgery* (prior to the first surgical procedure in the referral centre, 56 variables), *post-surgery* (posterior to the first surgical procedure and prior to ICU admission, 723 variables), *baseline* (BL; first 24 h of ICU admission, 762 variables). Figure 1a provides a graphical overview of data collection time points. Data cleaning, imputation, and pre-processing have been implemented as previously described [20]. For validation of models, the same subset of variables required for predictive models was extracted from the validation cohort and pre-processed using the same procedures.

#### Selection of input variables for the prediction

Relevant variables for predicting each clinical endpoint were selected using unsupervised variable selection with the Boruta algorithm [32]. An iterative version was implemented to obtain robust sets of relevant variables. Therein, we (i) created a subset of the dataset by randomly sampling 80% of patients with replacement, (ii) iteratively ran Boruta for 50 times with different initialisation on the bootstrapped dataset, and (iii) repeated steps (i)–(ii) for 30 iterations. Only variables identified in more than 50% of the iterations were considered relevant and kept for further analyses.

#### Machine-learning model development

A graphical overview detailing the development and validation process of the machine learning models can be found in Fig. 1b.

#### Model selection

For the prediction of binary endpoints (GAS aetiology, need for RRT, risk of amputation), we used a Random Forest classifier [33] as the predictive algorithm. For regression tasks (length of ICU stay, maximum size of skin defect), we compared multiple models including lasso regression, ridge regression, elastic nets, Gaussian process regression [34], multi-layer perceptron regression, and Random Forest regression [33].

Predictive power was compared through leave-one-out cross-validation. Model hyperparameters were optimised through an exhaustive grid search, using balanced accuracy for classification and negative mean squared error for regression as scoring metrics; a summary on the hyperparameters optimised can be found in Additional file 1: Note 3. Only the best performing models were used for subsequent internal validation with bootstrapping.

#### Model training and internal validation

Cross-validation of the model was conducted to ensure optimism-corrected performance evaluation and quantify the uncertainty associated with the developed models, in such a way that data and information leakage was prevented during the process of model training and hyperparameter optimisation. The entire process was repeated over *n* = 10^3^ bootstrapped samples of the development cohort data to obtain a measure of variability over the different data splits and enabling the calculation of robust 95% confidence intervals (95% CIs).

#### External validation of the prediction model

With the aim of testing a realistic scenario resembling the public deployment and subsequent external use of our model, we adopted the following external validation strategies: (1) the best performing model of the model obtained through the cross-validated selection process was fit to the whole development cohort (*n* = 409). Secondly, the generalisation performance of the trained models was assessed on bootstrapped samples from the validation cohort (*n* = 10^4^). Therein, the validation cohort was repeatedly resampled with replacement and predicted using the model, enabling the calculation of 95% CIs. (3) As an optional third step, we additionally assessed the adoption of a local validation scheme as proposed by Youssef et al. [35], which is intended to account for data heterogeneity across time, geography, and facilities by fine-tuning models trained on the development cohort using 30% of the validation data.

#### Model performance

The performance of classification models was evaluated using precision, recall, *F*_1_-score, balanced accuracy, Brier score [36], area under the receiver-operator curve (ROC-AUC), and average precision-recall score (PR). Regression model performance was assessed using the coefficient of determination (*R*^2^) as well as the mean absolute error (MAE) and mean squared error (MSE). Quality measures are given as mean and associated 95%CI calculated over all bootstrapping iterations. ROC-AUC is given only in the case of balanced groups as this measure can give a biased indication in the presence of unbalanced data.

#### Model explainability

SHapley Additive exPlanations (SHAP) values were used to assess the relative contribution of clinical variables to the prediction/classification models [37]. SHAP values were calculated for each model training/validation step since they are sensitive to model parameterisation and data splits. Results are given as mean with an associated 95% CI.

#### Software

For all machine-learning models, the implementations available in the scikit-learn Python library (version 1.4.2) were used. For variable selection, Boruta version 0.3 was used [32]. SHAP analysis was conducted using the “SHAP” package in Python version 0.43.0. Our source code for the GAS predictive models is available at https://github.com/sonjakatz/permit-nsti-gas and https://github.com/esaccenti/cdss_gas_nsti.

## Results

### Prediction of GAS aetiology in NSTI

The overarching goal was to predict GAS aetiology in NSTI as early as possible, as early assessment of aetiology is pivotal for deciding on NSTI treatment: we trained predictive models for the presence/absence of GAS using clinical variables recorded at different time points, namely *entry*,* pre-surgery*,* post-surgery* and *baseline* (see Fig. 1a and Table 1). We performed variable selection to identify a minimal set of relevant variables that are feasible to obtain in a clinical setting. This approach aimed to optimise the model by reducing the risk of overfitting while maintaining high prediction accuracy. The number of selected variables ranged from 6 (*entry*) to 14 (*baseline*), as shown in Table 1.

**Fig. 2:**
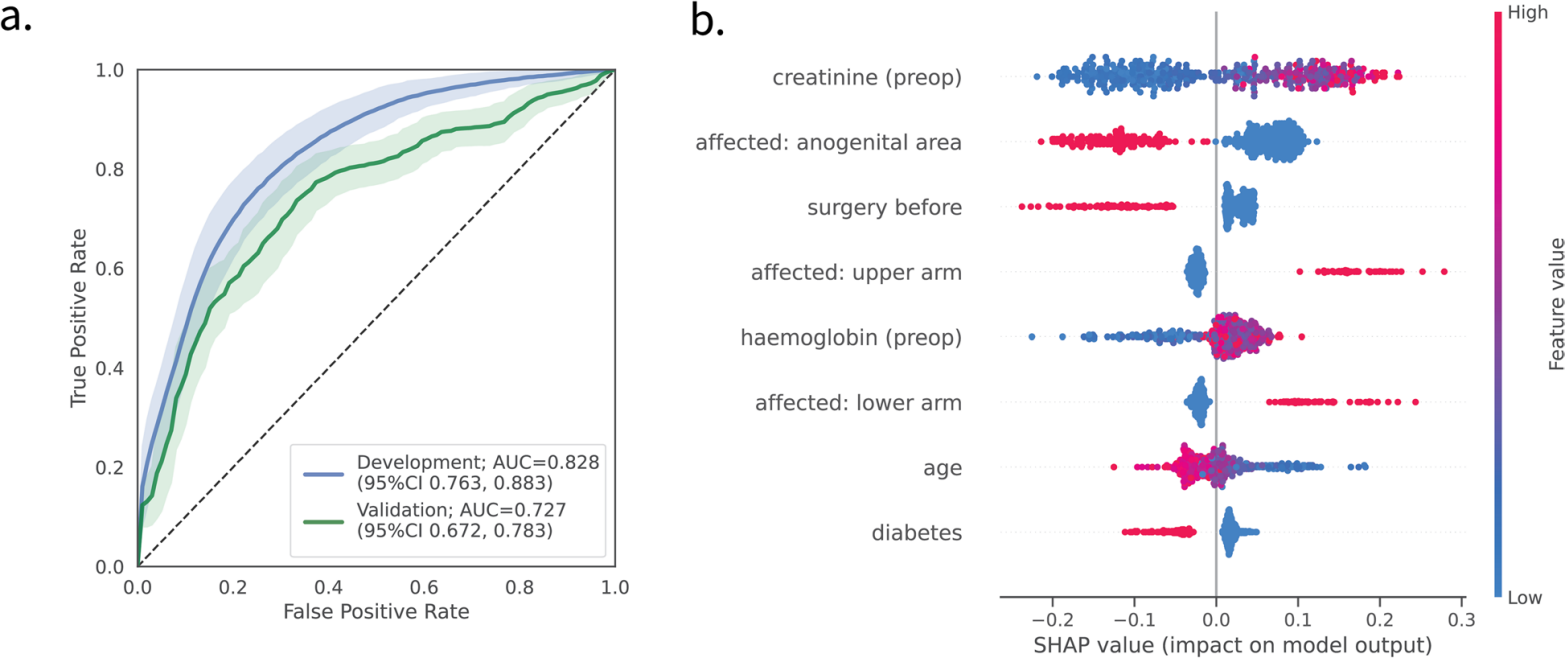
Performance of GAS prediction models. **a** ROC-curves for the models trained on development (blue) and external validation cohort (green) 95%CI: 95% confidence interval derived through 1000 (development) and 10,000 (validation) bootstrapped samples. **b** SHAP analysis for variable importance for models for the prediction of GAS aetiology. Variables are sorted from most impactful (top, creatinine) to least impactful (bottom, diabetes), with every dot representing a patient. Positive SHAP values for a variable indicate a positive contribution to the model’s decision to identify the patient as GAS-positive. Conversely, negative SHAP values indicate a contribution to classifying the patient as GAS-negative. The colour gradient denotes the variable values, with red indicating high values (e.g. age ~ 70 years) and blue indicating low values (e.g. age ~ 20 years). SHAP findings are consistent between development and validation cohort (see Additional file 1: Fig. S4)

As a next step, we assessed the earliest possible timepoint to predict GAS involvement. Therefore, we compared the prediction performances of the time-dissected datasets, ranging from *entry* (hospital admission) to *baseline* (first 24 h in the ICU) using a rigorous internal validation scheme (see the ‘Methods’ section). This revealed distinct differences between the predictive power of sets of variables collected at different time points (Table 2 and Additional file 1: Fig. S1). Notably, performances peaked using information already available *pre-surgery* (ROC-AUC 0.828; 95%CI [0.763, 0.883]), constituting the earliest possible time point for prediction of GAS aetiology (Fig. 2a). As timely intervention and revision of therapeutic regimen in patients showing GAS infection is of paramount clinical importance, we selected the *pre-surgery* for all further analyses.

**Table 2:** Model performances in estimating GAS involvement for time-dissected data. Depicted are the mean and the 95% confidence intervals (95%CI) [in parentheses] from internal validation, as well as external validation for the pre-surgery model (last column). *Acc.* balanced accuracy, *Prec.* precision, *Brier* Brier score, *ROC-AUC* area under the receiver-operator curve, *Av. prec.* average precision

	**Entry**	**Pre-surgery**	**Post-surgery**	**Baseline**	**Pre-surgery (external val.)**
Acc	0.652 [0.574, 0.735]	0.726 [0.642, 0.796]	0.723 [0.644, 0.791]	0.727 [0.658, 0.799]	0.670 [0.624, 0.718]
Prec	0.572 [0.435, 0.724]	0.666 [0.548, 0.784]	0.683 [0.556, 0.811]	0.729 [0.600, 0.867]	0.697 [0.615, 0.788]
Recall	0.463 [0.267, 0.698]	0.583 [0.391, 0.739]	0.564 [0.396, 0.720]	0.547 [0.396, 0.700]	0.476 [0.396, 0.560]
*F*1-score	0.502 [0.351, 0.632]	0.617 [0.483, 0.719]	0.613 [0.486, 0.714]	0.621 [0.500, 0.729]	0.565 [0.488, 0.637]
Brier	0.170 [0.142, 0.203]	0.152 [0.128, 0.183]	0.149 [0.125, 0.180]	0.147 [0.125, 0.172]	0.205 [0.181, 0.228]
ROC-AUC	0.794 [0.733, 0.851]	0.828 [0.763, 0.883]	0.836 [0.775, 0.891]	0.839 [0.779, 0.894]	0.727 [0.672, 0.783]
Av. precision	0.617 [0.494, 0.719]	0.684 [0.568, 0.787]	0.685 [0.570, 0.788]	0.703 [0.592, 0.807]	0.666 [0.594, 0.736]

Next, we sought to validate the performance of the *pre-surgery* model in an external validation cohort, composed of 208 patients with information available on microbial aetiology (208/216 patients, 96.3%). A comparison of the variable characteristics between the development and validation cohorts showed a high degree of similarity (Table 3).

**Table 3:** Overview of selected predictive variables’ characteristics between the development and validation cohort. Only variables relevant for the *pre-surgery* model were considered. Detailed variable descriptions can be found in Additional file 1: Table S2. (%) indicates percentage relative to the total number of patients in the two cohorts

	**Development cohort** (*n* = 409)	**Validation cohort** (*n* = 208)
GAS, *n* (%)	126 (30.8)	82 (39.4)
Age, years, mean (SD)	58.7 (15.1)	58.2 (15.5)
Affected: upper arm, *n* (%)	48 (11.7)	20 (9.3)
Affected: lower arm, *n* (%)	56 (13.7)	23 (10.7)
Affected: anogenital area, *n* (%)	143 (35.0)	64 (29.8)
Surgery before, *n* (%)	77 (18.8)	53 (26.2)
Diabetes, *n* (%)	98 (24.0)	58 (26.9)
Creatinine (preop), mean (SD)	161.2 (121.3)	153.7 (123.3)
Haemoglobin (preop), mean (SD)	11.4 (3.04)	12.7 (2.5)

Overall, the model showed a good discriminatory power within the validation cohort: ROC-AUC = 0.727, 95%CI [0.672, 0.783], (see Fig. 2a, green line for the ROC curve).

#### Fine tuning and local validation

We additionally evaluated the performance of a localised version of the validation model by fine-tuning trained models using 30% of the validation data, which is intended to account for data heterogeneity across time, geography, and facilities. This improved the performance to a ROC-AUC = 0.758, 95%CI [0.696, 0.821] (see Additional file 1: Fig. S2, pink lines for ROC curve). Determination of the optimal threshold by trying to minimise the number of false negatives yielded an ideal cutoff value of 30%, resulting in a good trade-off between sensitivity and specificity (Table 4).

**Table 4:** Summary of the accuracy of external validation (pre-surgery) model at different decision threshold levels. Means are given together with the associated 95% confidence intervals [in parentheses]. Highlighted in bold is the threshold identified as the optimal trade-off between model precision and recall when aiming to reduce false negatives as much as possible. The FPR can be interpreted as the risk of over-treatment, while the FNR indicates the risk for under-treatment. *TPR* true positive rate, *TNR* true negative rate, *FPR* false positive rate, *FNR* false negative rate

Threshold	Sensitivity (TPR)	Specificity (TNR)	FPR	FNR
0	1.00 [1.00, 1.00]	0.00 [0.00, 0.00]	1.00 [1.00, 1.00]	0.00 [0.00, 0.00]
0.1	0.97 [0.94, 0.99]	0.08 [0.03, 0.11]	0.92 [0.88, 0.96]	0.03[0.00, 0.04]
0.2	0.79 [0.73, 0.86]	0.44 [0.39, 0.48]	0.56 [0.49, 0.63]	0.21 [0.14, 0.27]
**0.3**	**0.62 [0.57, 0.67]**	**0.67 [0.63, 0.72]**	**0.32 [0.26, 0.38]**	**0.39 [0.31, 0.48]**
0.4	0.40 [0.37, 0.44]	0.85 [0.82, 0.88]	0.14 [0.09, 0.19]	0.60 [0.52, 0.68]
0.5	0.26 [0.23, 0.30]	0.95 [0.93, 0.97]	0.05 [0.02, 0.07]	0.75 [0.67, 0.82]
0.6	0.21 [0.18, 0.24]	1.00 [0.98, 1.00]	0.01 [0.00, 0.01]	0.79 [0.73, 0.86]
0.7	0.05 [0.03, 0.07]	1.00 [0.98, 1.00]	0.01 [0.00, 0.01]	0.95 [0.92, 0.98]
0.8	0.00 [0.00, 0.00]	1.00 [1.00, 1.00]	0.00 [0.00, 0.00]	1.00 [1.00, 1.00]
0.9	0.00 [0.00, 0.00]	1.00 [1.00, 1.00]	0.00 [0.00, 0.00]	1.00 [1.00, 1.00]
1	0.00 [0.00, 0.00]	1.00 [1.00, 1.00]	0.00 [0.00, 0.00]	1.00 [1.00, 1.00]

### Model interpretation

To gain insight into the model’s decision-making and to quantify the contribution of individual variables, we conducted post-hoc interpretability analysis using SHapley Additive exPlanations (SHAP) (Fig. 2b). SHAP values revealed that anatomical location significantly influenced model decisions: infections in upper extremities were highly predictive of GAS, while anogenital infections suggested non-GAS aetiology.

Diabetes, recent surgery (within 4 weeks), and age over 50 years indicated a lower risk of GAS while preoperative creatinine levels above 110 µmol/L were the most influential variable for predicting GAS (Additional file 1: Fig. S3a–c).

SHAP findings were consistent across the development and validation predictive models (Additional file 1: Fig. S4).

### Predicting clinical endpoints: surgical aspects, patient management, and need of organ support

We further explored the possibility of predicting several endpoints of clinical relevance using the same sets of variables recorded at different time points (see Fig. 1a) and in the case of GAS prediction. We trained predictive models for surgical endpoints (risk of amputation, the size of skin defect after fist surgery, the maximal size of skin defect), patient management (length of ICU stay), as well as the necessity of organ support (need for RRT within 24 h after ICU (BL)).

**Fig. 3:**
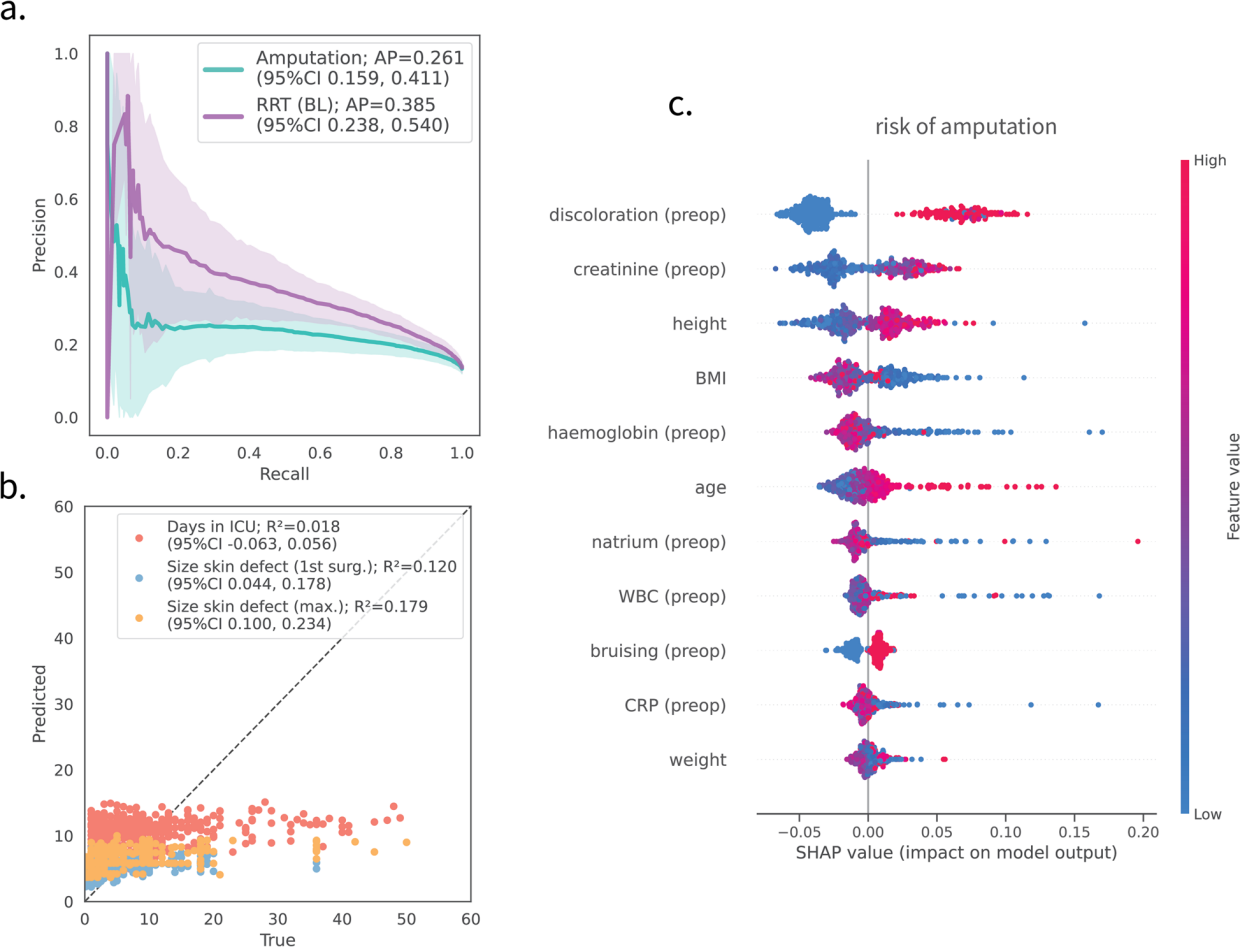
Prediction performance for clinical endpoints revolving around surgical aspects, patient management, and organ support. **a** Precision-recall curves for binary endpoints, including predicting the risk of amputation (turquoise) and the need for RRT within the first 24 h after ICU admission (baseline, BL). **b** Predicted versus true values for continuous endpoints, including the estimated days in ICU (red, days), the size of the skin defect after the first surgery (blue, percent body surface), and the maximal size of skin defects (orange, percent body surface). **c** SHAP values for models predicting the risk of amputation. The mean and the 95% confidence intervals derived through 1000 bootstrapped samples are given. Summary on included variables and more performance metrics can be found in Additional file 1: Tables 3 and 4, respectively. AP: average precision, *R*^2^ coefficient of determination

For each outcome, unsupervised variable selection yielded between 11 and 16 predictive variables (Additional file 1: Table 4). Internal validation of the best performing models revealed a lack of predictive performance across all clinical endpoints (Fig. 3a, b).

Analysis of SHAP values for surgery-related endpoints, such as the risk of amputation, highlighted the importance of clinically relevant variables like discoloration, creatinine values, and patient age (Fig. 3c).

## Discussion

In this study, we developed and externally validated models for the prediction of GAS aetiology and the assessment of clinical endpoints related to NSTI. Results show the possibility of successfully predicting GAS aetiology using a set of eight variables recorded before first surgery in the referral centre. Results were confirmed by deploying the model on an external cohort of NSTI patients, addressing the pivotal task of establishing the aetiology of the infection. Prediction of other clinical endpoints was not successful. 

### GAS aetiology in INSTI

NSTIs are classified by heterogeneity in causal microbial aetiology, categorised as polymicrobial infections called type 1, and monomicrobial infections, called type 2 [38]. The most frequent cause of type 2 infections is GAS, causing 15–40% of cases. Data support thats there is a slow increase in incidence rates of all NSTIs [39].

In contrast, the incidence of severe GAS infection is fluctuating over time. Since 2022, there has been a worldwide historic upsurge in invasive (iGAS) disease [40]. NSTI caused by GAS most frequently leads to septic shock, multi-organ failure, and the need for treatment at the intensive care unit (ICU) level [41]. In a recent French study of post-pandemic iGAS treated in ICUs, NSTI was the most frequent cause of infection, comprising 44% of cases [42].

In NSTI, patient number is low, and identification is challenging in the initial stages of the disease, leaving patients at risk for treatment delay or receiving incomplete measures. Several clinical score systems to aid early diagnosis of NSTI have been proposed, but controversies remain [3, 4, 41, 43]. Today, preoperative methods to corroborate GAS aetiology in NSTI pre-surgery are not available. Gram staining of tissue samples obtained during surgery is one of the quickest methods for substantiating streptococcal involvement in NSTI. Intraoperative use of a rapid antigen detection test for identification of GAS aetiology has been studied, but these tests are not validated for use on soft tissue, and data is still scarce and quality disputable [44].

Challenges and controversies on optimal treatment of NSTI still remain, in which early diagnosis may add value to decisions. In patients with GAS NSTI, surgery is performed at an earlier stage if compared to patients showing type 1 infections [30, 41]. Guidelines recommend surgery within 6–12 h [45], but even in the INFECT study, the largest study of NSTI to date with prospective patient enrolment and low mortality rates, median time to surgery in GAS aetiology was 16 h [41].

Data lends support to the inclusion of clindamycin from the start of antibiotic therapy, which is reflected in contemporary treatment guidelines [45]. Clindamycin is given primarily to inhibit toxin production by GAS [45]. Clindamycin resistance in GAS is increasingly frequent [46], calling for early awareness of the risk of insufficient treatment effect which could potentially be provided by early assessment of GAS aetiology from variables collected at initial stages, as shown in this study. Furthermore, several GAS *emm*-types hold the potential to form communities producing biofilm in NSTI [24, 25], calling for awareness about treatment efficacy. In GAS NSTI, there is data to support treatment with intravenous immunoglobulin (IVIG) [41, 47].

The eight variables selected for predicting GAS through unsupervised variable selection were diverse, ranging from the anatomical location of the infection to standard laboratory measurements. Despite the diversity, all the selected variables can easily be obtained in routine practice in a standard hospital setting and do not require additional examinations or tests.

Prior research in NSTI has shown a higher occurrence of monomicrobial infections, including GAS, in the upper extremities, contrasting with polymicrobial infections more common in the truncal region [30, 48]. Our study found a negative association between other surgeries performed in the last 4 weeks prior to the NSTI and GAS aetiology.

In the INFECT study, 77/409 patients (19%) had undergone recent surgery [30], compared to 5/126 (4%) in patients with GAS aetiology [41]. Also, GAS NSTI cases showed elevated creatinine, and were more frequently associated with septic shock (65%) and multiorgan dysfunction compared to NSTI of other microbial aetiologies [41]. Conversely, GAS NSTI cases showed a negative association with haemoglobin concentrations, with no red blood cell transfusions observed in the entire cohort [30]. This may be attributed to higher rates of pre-existing comorbidities, longer clinical courses, and more recent surgeries in non-GAS cases [30, 41].

In invasive GAS infection, higher age-adjusted incidence rates are seen among the elderly [49, 50]. This is also the case for NSTI of any reason (type 1 and type 2 altogether) [51, 52]. However, we showed increased likelihood of GAS aetiology in the patients with less advanced age in this infection category. Similarly, recent surgery is an independent risk factor in NSTI, but primarily in type 1 infections: it is a rare event in GAS NSTI cases, thereby adding value to the model.

Altogether, in the case of GAS aetiology, there may be added value for the handling of patients by early identification of causal microbial agents, motivating early initiation of individualised therapy and other measures.

### IVIG treatment in NSTI

Since treatment with IVIG is costly, the benefits of earlier initiation of treatment should be balanced with the potential risk of overtreatment of patients with non-GAS NSTI. Therefore, we suggest using the 0.3 threshold (Table 4) to be used in clinical practice. This could lead to the correct early identification of 67% of patients with GAS aetiology, while leading to 31% overtreatment (false positives). Although a lower threshold would improve correct identification, it can also substantially increase overtreatment.

### Fine tuning and local validation of GAS prediction model

We also explored the possibility of employing local validation through fine tuning as an alternative to external validation, as step recurring local validation to guarantee that the prediction models remain valid and to protect against the effect of data variability: this showed that fine-tuning trained models on a fraction of the validation data further improved model performances without leading to over-fitting. The rationale here is to define a bias between the generalisability of the model and the maximisation of its efficacy within a particular local clinical setting while retaining usefulness and fairness [35].

### Prognostication and prediction of clinical endpoints in NSTI

Most studies on prognostication and prediction of clinical endpoints in NSTI focus on mortality and amputation [30, 53–58]. Predictions of other clinically relevant endpoints [20], such as the size of the skin defect post-surgery [31], ICU length of stay [59], and the need for Renal Replacement Therapy (RRT) [60, 61], have received less attention, especially not for patients with NSTI. In NSTI, there is no universal approach for how to perform surgical debridement in the acute phase. Commonly, decisions on surgical approach are demanded of the surgeon, reflected in variable resection practice as also seen in the two cohorts in this study. This is likely to influence other outcomes beyond surgical aspects. It is therefore expected that we were unable to establish prediction models for these outcomes, as widely accepted survival guidelines must be in place to lower the impact of individual approaches in surgical treatment.

Our assessment of surgical endpoints, such as the risk for amputation and the size of the skin defect, underscores the challenge in objectively evaluating these endpoints. Notably, variables crucial for predicting amputation risk—such as discoloration, bruising, or patient age—appear to be linked to clinical aspects of surgical outcomes [62]. However, despite their clinical relevance, these variables alone do not adequately predict skin defect size or the likelihood of extremity amputation. This suggests that the decisions regarding amputation and the extent of skin excision are subjective and influenced by individual surgeons or local guidelines and do not necessarily reflect molecular, clinical, or epidemiologic parameters directly obtained from patients data.

A recent survey on NSTI debridement practices among Dutch general and plastic surgeons supports this hypothesis, revealing significant differences in the amount of skin resection deemed necessary [63]. These results indicate a similar variability in the decision-making process surrounding the need for amputation. Moreover, genetic predisposition in the STING gene has been linked to amputation and associated with the expression of virulence factors, indicating the complex interaction between host and pathogen influencing NSTI patients’ outcomes [64].

Solely using clinical information ranging from hospital admission to the first 24 h in ICU, we were also unable to accurately estimate the length of stay in the ICU. Given the complexity and varied progression of NSTI, patients often require prolonged ICU stays [30], therefore, incorporating more longitudinal ICU data appears essential for improving prediction accuracy.

Although previous studies have shown the predictive value of assessing renal status in determining the need for renal replacement therapy [60, 61], the limited number of RRT cases in the INFECT cohort resulted in wide confidence intervals: this hindered our ability to conclusively evaluate the effectiveness of our models in predicting RRT.

### Limitations of the study

The study’s strength lies in combining clinical and computational biology expertise to investigate previously unexplored clinically significant endpoints for NSTI. Leveraging the largest available NSTI cohort, we both conducted rigorous internal validation and externally validated the predictive models using data from a referral centre not included in the original study. This approach allowed us to realistically assess the generalisability of the predictive models.

However, recent concerns about the clinical utility and generalisability of externally validated models across different institutions have been raised [35, 65]. By fine-tuning pre-trained models with local data, we demonstrated the importance of local adaptation, ensuring models can accommodate heterogeneity across times, locations, and facilities without overfitting.

The strengths of our findings should be considered within the context of certain limitations. The development cohort used originates from an ICU-focused study, with limited access to pre-hospital data. Due to the uncertainty surrounding the timing of initial symptoms, there exists the possibility of considerable diversity in the progression of the disease among patients, which could potentially affect the performance of the models. Also, we believe the inability to estimate patient management and need of organ support can be partially attributed to the lack of longitudinal data and large imbalances in target labels. Lastly, despite successful external validation, the clinical utility of our models warrants further assessment through prospective validation directly comparing model predictions with clinician decisions.

## Conclusions

In this study, we demonstrate the successful early prediction of GAS aetiology using minimal patient data, suggesting the potential for targeted interventions earlier in the NSTI disease course. The early availability of predictive variables implies that our models could be potentially used in an emergency room setting. Given the rising incidence of GAS in the Western world [66–68], and studies indicating the beneficial effects of early IVIG administration on survival in GAS patients [41], we believe our findings hold high clinical relevance. Additionally, our conclusions drawn from the prediction of clinical endpoints highlight the need for more research on surgical decision-making. The approach in this study may be used for relevant predictions in other severe infections too.

To the best of our knowledge, this study represents the first use of machine learning to estimate NSTI aetiology and relevant clinical endpoints. Using only eight readily available variables, we developed and validated models capable of estimating the bacterial aetiology prior to surgical debridement. We believe the results of this study to have significant implications for sepsis treatment in patients with NSTI caused by GAS, which may improve their survival and quality of life, enhance management of household contacts and reduce the risk of hospital spread of GAS.

## Supplementary Information

Additional file 1.

## Data Availability

The datasets generated and/or analysed during the current study are not publicly available as they include sensitive personal health data but are available reasonable request given national regulations from E.S ([edoardo.saccenti@wur.nl](mailto:edoardo.saccenti@wur.nl)) and A. N-T. (anna.norrby-teglund@ki.se) for what concerns the INFECT training data and from A. dV. ([adevries@rkz.nl](mailto:adevries@rkz.nl)) or A.P. ([apijpe@rkz.nl](mailto:apijpe@rkz.nl)) for the Dutch validation data.

## Abbreviations

BL: Baseline
GAS: *Streptococcus **pyogen*Es (Group A *Streptococcus*)
LOOCV: Leave-one-out cross-validation
MAE: Mean absolute error
MSE: Mean squared error
NSTI: Necrotising soft tissue infections
PR: Precision–recall (average precision–recall score)
RRT: Renal replacement therapy
ROC-AUC: Receiver-operator curve area under the curve
*R*^2^: Coefficient of determination
SAPS II: Simplified Acute Physiology Score II
SHAP: SHapley Additive exPlanations
SOFA: Sequential Organ Failure Assessment
