# Supplementary material for: A validated model for early prediction of group A streptococcal aetiology in necrotising soft tissue infections using minimal patient data
Source: BMC Med. 2026 Jan 10;24:76. doi: 10.1186/s12916-025-04593-y (PMC12882190; doi:10.1186/s12916-025-04593-y)
Supplement: Supplementary file 1 — Additional file 1. [file 12916_2025_4593_MOESM1_ESM.pdf]

A validated model for early prediction of group A streptococcal aetiology in necrotising soft tissue infections using minimal patient data

Additional Material

Sonja Katz[1,2], Jaco Suijker[3,4,5], Steinar Skrede[6,7], Annebeth Meij-de Vries[3,8], Anouk Pijpe[3,5], Anna Norrby-Teglund[9], Laura M Palma Medina[9], Jan K Damås[10,11,12], Ole Hyldegaard[13,14], Erik Solligård[10,15], Mattias Svensson[9], PerAID/PerMIT/INFECT study group[†], Knut Anders Mosevoll[6,7], Vitor AP Martins dos Santos[16,17], Edoardo Saccenti[1]*

*Corresponding author.
Edoardo Saccenti, Systems & Synthetic Biology, Stippeneng 4, 6708WE Wageningen, The Netherlands, edoardo.saccenti@wur.nl

[1] Laboratory of Systems and Synthetic Biology, Wageningen University and Research, Wageningen, The Netherlands
[2] Department of Radiology and Nuclear Medicine, Erasmus MC, Rotterdam, The Netherlands.
[3] Alliance of Dutch Burn Care, Red Cross Hospital, Vondellaan 13, 1942 LE, Beverwijk, The Netherlands
[4] Amsterdam UMC location Vrije Universiteit Amsterdam, Plastic, Reconstructive and Hand Surgery, De Boelelaan 1117, Amsterdam, The Netherlands
[5] Amsterdam Movement Sciences, Tissue Function and Regeneration, Amsterdam, The Netherlands
[6] Department of Clinical Science, University of Bergen, Pb 7804, 5020, Bergen, Norway
[7] Department of Medicine, Haukeland University Hospital, Pb 1400, 5021, Bergen, Norway
[8] Pediatric Surgical Centre, Emma Children's Hospital, Amsterdam UMC, Amsterdam, the Netherlands
[9] Center for Infectious Medicine, Karolinska Institutet, Karolinska University Hospital, Huddinge, Sweden

Commented [SS1]: Please note that in Supplementary Material, Jan Kristian is included in list of non-authors involved. To be corrected there.

[10]Mid-Norway Centre for Sepsis Research, Department of Circulation and Medical Imaging, Norwegian University of Science and Technology

[11] Department of Infectious Diseases, St. Olav's Hospital, Trondheim University Hospital

[12] Centre of Molecular Inflammation Research, Department of Clinical and Molecular Medicine, Norwegian University of Science and Technology, Trondheim, Norway

[13] Department of Clinical Medicine, University of Copenhagen, Copenhagen, Denmark

[14] Department of Anaesthesia, Centre of Head and Orthopaedics, Copenhagen University Hospital, Copenhagen, Denmark

[15] Norwegian University of Science and Technology NO

[16] Department of Bioprocess Engineering, Wageningen University & Research, Wageningen WE, The Netherlands

[17] LifeGlimmer GmbH, Berlin, Germany

[†] The PerAID/PerMIT/INFECT consortium members are listed in **Additional file 1: Note 1.**

Supplementary Note 1

The PerAID/PerMIT/INFECT consortium

Lead investigators:

Coordinating investigator

Anna Norrby-Teglund
Centre for Infectious Medicine, Department of Medicine Huddinge
Karolinska Institute, Stockholm, Sweden

National site investigators

Copenhagen
Ole Hyldegaard
Department of Anaesthesia, Centre of Head and Orthopaedics
Copenhagen University Hospital, Rigshospitalet, Copenhagen, Denmark

Stockholm
Michael Nekludov
Department of Anaesthesia, Surgical Services and Intensive Care
Karolinska Institute, Karolinska University Hospital, Stockholm, Sweden

Karlskrona
Ylva Karlsson
Department of Anaesthesia and Intensive Care
Blekinge County Hospital, Karlskrona, Sweden

Gothenburg
Per Arnell
Department of Anaesthesia and Intensive Care
Sahlgrenska University Hospital, Gothenburg, Sweden

Bergen
Steinar Skrede
Department of Medicine
Haukeland University Hospital, Bergen, Norway
Department of Clinical Science,
University of Bergen, Bergen, Norway

The INFECT study group:

Karolinska Institutet, Karolinska University Hospital, Stockholm Sweden
Anna Norrby-Teglund (Co-ordinator), Mattias Svensson (Project Manager), Muhammad Afzal, Helena Bergsten, Lydia Bosnak, Bavya Chakrakodi, Puran Chen, Johanna Emgård, Linda Johansson, Julius Juarez, Srikanth Mairpady Shambat, Nikola Siemens, Johanna Snäll, Julia Uhlman, Takeaki Wajima.

Copenhagen University Hospital, Rigshospitalet, Copenhagen, Denmark
Ole Hyldegaard (Team Leader, Co-ordinator clinical partners), Martin B. Madsen (Clinical Database), Daniel Bidstrup, Nina F. Bærnthsen, Julie V. Clausen, Anna Damgaard, Gladis H. Frendø, Martin Forchammer, Marco Hansen, Morten FF Hedetoft, Karen L. Hilsted, Diana Isaksen, Erik C. Jansen, Josefine Kofoed, Anette Lilja, Lærke B. Madsen, Rasmus Müller, Isabel S. Nielsen, Emilie MJ Pedersen, Marie W. Petersen, Anders Perner, Peter V. Polzik, Frederikke Ravn

Karolinska University Hospital, Stockholm, Sweden
Michael Nekludov (Team Leader), Folke Lind, Anders Kjellberg, Erik von Oelreich, Peter Kronlund, Sverre Kullberg, Ola Friman, Lisa Hellgren, Anna Granström, Anna Schenning, Sandra Carlsson

Haukeland University Hospital, Bergen, Norway
Steinar Skrede (Team Leader), Trond Bruun, Torbjørn Nedrebø, Oddvar Oppegaard, Eivind Rath, Marianne Søvik, Hanne Søyland

Blekingesjukhuset Karlskrona
Ylva Karlsson (Team Leader), Dag Benoni

Sahlgrenska University Hospital Ostra, Gothenburg, Sweden
Per Arnell (Team Leader), Hans Lycke, Joakim Trogen, Kerstin Ohlauson

Helmholtz Center for Infection Research, Braunschweig, Germany
Dietmar H. Pieper (Team Leader), Singh Chhatwal, Andreas Itzek, Anshu Babbar, Robert Thänert, Jörn Hoßmann, Eva Medina, Domenica Hamisch, Israel Barrantes, Patric Nitsche-Schmitz, Astrid Dröge, Katja Mummenbrauer.

Wageningen University and Research, Wageningen, The Netherlands
Vitor Martins Dos Santos (Team Leader), Edoardo Saccenti

Université Lyon 1, Lyon, France
Francois Vandenesh (Team Leader), Sylvere Bastien, Jessica Baude, Anne Tristan.

LifeGlimmer GmBH, Berlin, Germany

Vitor Martin dos Santos (Team Leader), Erno Lindfors, Francois Bergey

Cube Dx GmbH, Sankt Valentin, Austria
Christoph Reschreiter (team Leader), Bernhard Ronacher, Matthias Pilecky

Tel Aviv University, Tel Aviv, Israel.
Eytan Ruppin (Team Leader), Matthew Oberhardt, Raphy Zarecky.

University of North Dakota, Grand Forks, USA
Malak Kotb (Team Leader), Karthickeyan Chellakrishnan, Santhosh Mukundan, Suba Nokala,

The Lee Spark NF Foundation, UK

Doreen Marsden (Team Leader).

PerAID/PerMIT Study Group - non-authors involved

Mattias Svensson[1], Kristoffer Strålin[1,2], Trond Bruun[3,4], Oddvar Oppegaard[3,4], P.P.M van Zuijlen[5,6,7], Marco Anteghini[8,9]

1 Center for Infectious Medicine, Department of Medicine Huddinge, Karolinska Institute, Stockholm, Sweden
2 Department of Medicine, Huddinge, Karolinska Institutet, Stockholm, SWEDEN
3 Department of Medicine, Haukeland University Hospital, Bergen, Norway
4 Department of Clinical Science, University of Bergen, Bergen, Norway
5 Burn Centre, Department of Plastic, Reconstructive and Hand Surgery, Red Cross Hospital, Beverwijk, The Netherlands
6 Pediatric Surgical Centre, Emma Children's Hospital, Amsterdam UMC, Amsterdam, The Netherlands
7 Department of Plastic, Reconstructive and Hand Surgery, Amsterdam Movement Sciences Amsterdam UMC, Amsterdam, The Netherlands
8 LifeGlimmer GmbH, Berlin, Germany
9 Laboratory of Systems and Synthetic Biology, Wageningen University and Research, Wageningen, The Netherlands

Supplementary Note 2

Dutch Centres of investigation (Validation cohort)

		Location	Academic	Burn centre
1	Amsterdam UMC loc. VUmc	Amsterdam	Yes	No
2	Amsterdam UMC loc. AMC	Amsterrdam	Yes	No
3	LUMC	Leiden	Yes	No
4	Erasmus MC	Rotterdam	Yes	No
5	Red Cross Hospital	Beverwijk	No	Yes
6	Maasstad Hospital	Rotterdam	No	Yes
7	Martini Hospital	Groningen	No	Yes
8	Noordwest Ziekenhuisgroep	Alkmaar	No	No
9	OLVG	Amsterdam	No	No
10	Spaarne Gasthuis	Hoofddorp	No	No
11	BovenIJ ziekenhuis	Amsterdam	No	No

Supplementary Note 2

Overview of Tuned Machine Learning Hyperparameters

Classification algorithms:

Logistic Regression (LR):

- *penalty*: none, l2
- *C*: 0.001,0.01,0.1,1,10,100,1000

Gaussian process classifier (GPC):

- *kernel*: DotProduct, Matern, RationalQuadratic, WhiteKernel

Gaussian Naive Bayes (GNB):

- *var_smoothing*: 100 steps from -9 until +9

Random Forest Classifier (RFC):

- *n_estimators*: 100, 300, 700
- *max_depth*: 2,4,6
- *max_features*: 2,4,6

Regression algorithms:

Lasso regression:

- *selection*: “random”
- *alpha*: 1.0, 0.7, 0.5

Ridge regression

- *alpha*: 1.0, 0.7, 0.5

Elastic nets:

- *l1_ratio*=0.5
- *selection*="random"
- *alpha*: 1.0, 0.7, 0.5

Multi-layer perceptron regression:

- *activation*: "relu"
- *batch_size*: 64
- *early_stopping*: True
- *solver*: “adam”
- *alpha*: 1, 0.1, 0.01, 0.001, 0.0001

- *hidden_layer_size*: (10, 3), (11, 3), (8, 3), (7, 3), (9, 3)

Random Forest Regressor:

- *n_estimators*: 100, 300
- *max_depth*: 2,4
- *max_features*: 2,4

Supplementary Figure 1.

Comparison performance between different time-dependent datasets for the estimation of the presence of GAS. (left) ROC curve (right) Precision-Recall curve. ENTRY (upon hospital admission), PRESURGERY (prior to first surgical procedure), POSTSURGERY (posterior to first surgical procedure and prior to ICU admission), BL (baseline; first 24 h of ICU admission). AUC: Area under the ROC curve, AP: average precision.

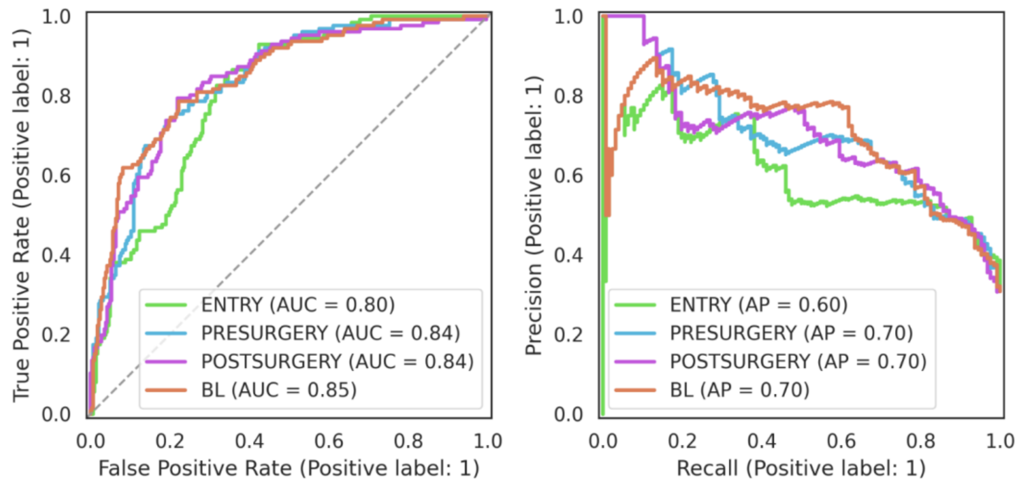

Supplementary Figure 2.

ROC-curves comparing the performance of the **development** (blue) and **external validation** (green), and **fine-tuned external validation** (pink). 95%CI: 95% confidence interval derived through 1000 (development) and 10000 (validation) bootstrapped samples

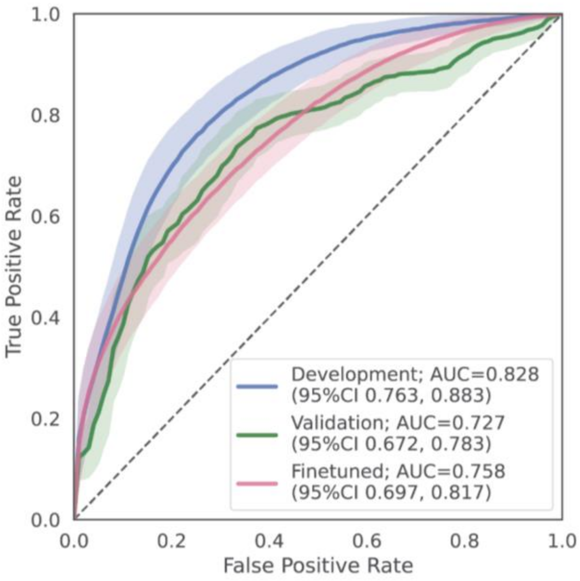

Supplementary Figure 3.

SHAP dependence plots for (a) age (b) creatinine (c) haemoglobin. The colour gradient denotes the variable values, with red indicating high values (e.g. age ~ 70 years) and blue indicating low values (e.g. age ~ 20 years).

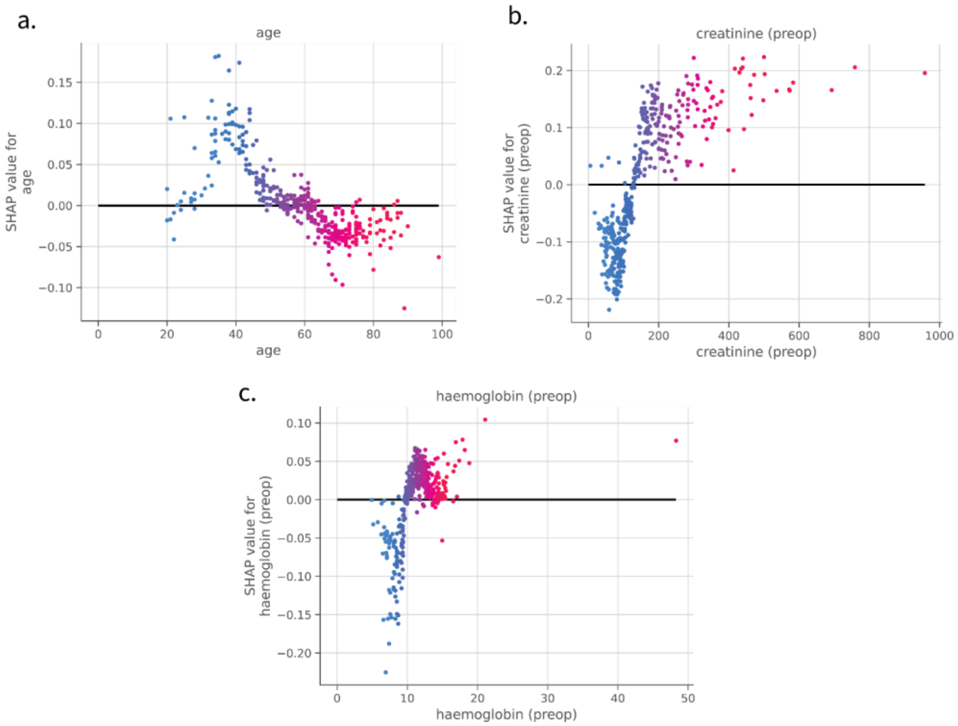

Supplementary Figure 4.

SHAP values for models estimating the presence of GAS with the external validation cohort. Variables are sorted from most impactful (top, creatinine) to least impactful (bottom, diabetes), with every dot representing a patient. Positive SHAP values for a variable indicate a positive contribution to the model's decision to identify the patient as GAS-positive. Conversely, negative SHAP values indicate a contribution to classifying the patient as GAS-negative. The colour gradient denotes the variable values, with red indicating high values (e.g. age ~ 70 years) and blue indicating low values (e.g. age ~ 20 years).

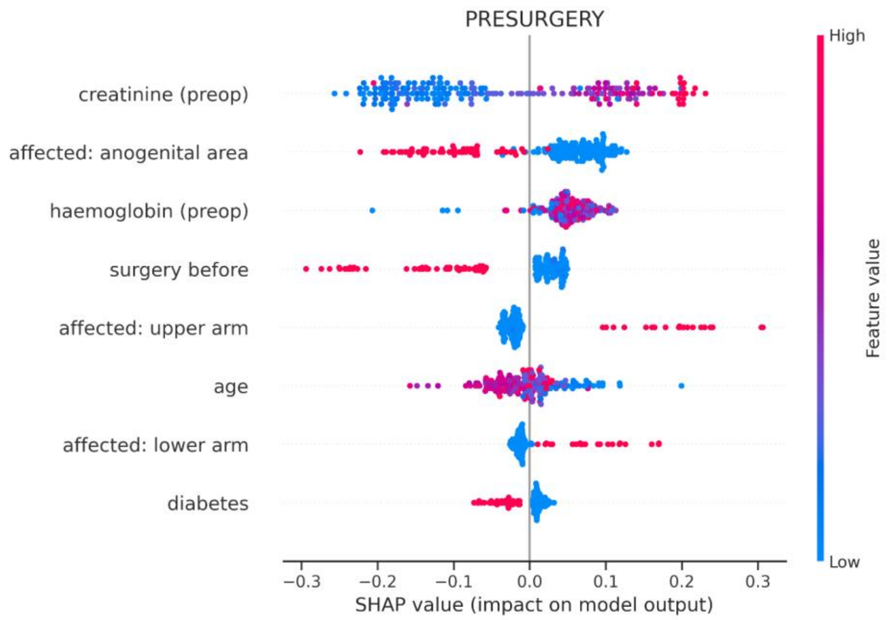

Supplementary Table 1.

Overview of the number of patients included and data subsets assessed for each clinical outcome.

Outcome category	Outcome	No. of patients (n)	Time-dependent data subsets	Prediction task	No. of patients (%) or mean ± SD
causative microbes	Presence of GAS	409	Entry, pre-surgery, post-surgery, baseline	classification (y/n)	126 (30.8%)
surgical aspects	Risk of amputation	409	Entry, pre-surgery	classification (y/n)	54 (13.2%)
	Size of skin defect (after first surgery)	391	Entry, pre-surgery	regression (pct. of body surface)	4.9 ± 5.2
	Size of skin defect (maximal)	409	Post-surgery, baseline	regression (pct. of body surface)	6.4 ± 7.3
patient management	Length of ICU stay	402	Entry, pre-surgery, post-surgery, baseline	regression (days)	10.7 ± 10.8
organ support	Need for RRT (within 24h after ICU admission)	409	Entry, pre-surgery, post-surgery	classification (y/n)	57 (13.9%)
	Need for RRT (within 90 days)	351	Baseline	classification (y/n)	24 (6.8%)

Supplementary Table 2

Detailed description for variables used in the estimation of the presence of GAS.

Variable name	Description
age	age at admission [years]
affected: upper arm	affection of upper arm[1] [y/n]
affected: lower arm	affection of lower arm[1] [y/n]
affected: anogenital area	affection of anogenital area[1] [y/n]
surgery before	surgery within 4 weeks previous of NSTI [y/n]
diabetes	diabetes [y/n]
creatinine (preop)	Highest preoperative[2] creatinine [μmol/L]
haemoglobin (preop)	Lowest preoperative[2] haemoglobin [mmol/L]
creatinine (preadmission)	Highest preadmission[3] creatinine [μmol/L]
Lowest systolic BP (BL)	Lowest BL[4] systolic blood pressure [mmHg]
Creatinine (BL)	Highest BL[4] creatinine [μmol/L]
Noradrenaline (BL)	Highest noradrenaline infusion rate at BL[4] [μg/kg/min]
Platelets (BL)	Lowest BL[4] platelets [10^9/L]
Lactate (BL)	Highest BL[4] lactate level [mmol/L]
Glucose (BL)	Highest BL[4] glucose [mmol/L]
Anatomical site sampled	Anatomical site[5] specimen was sampled from

[1] at arrival at sepcialized hospital

[2] before the first surgery, which is before ICU admission

[3] upon ICU admission

[4] during the first 24 hours in the ICU

[5] one of the following: head/neck, u. arm, l. arm, hand, finger, thorax, abdomen, ano-gen., u. leg, l. leg, foot, to

Supplementary Table 3.

Overview of variables yielded through unsupervised variable selection for prediction of surgical, patient management, and organ support outcomes. BMI: Body Mass Index, BL: baseline (24 hours after ICu admission), CRP: c-reactive protein, preop: preoperative, KDIGO: Kidney Disease Improving Global Outcomes, WBC: white blood cell count,

**Risk of amputation**	**Size of skin defect (after first surgery)**	**Size of skin defect (maximal)**	**Days spent in ICU**	**Need for RRT (24h after ICU admission)**	**Need for RRT (90 days)**
Weight	Weight	BP (highest BL)	No. of blood samples taken	Weight	Pulse (lowest BL)
Height	Height	Bilirubin (highest BL)	Discoloration (preop)	Height	Carbamid (highest BL)
Discoloration (preop)	Bullae (preop)	No. of blood samples (1st surgery)	WBC (preop)	Discoloration (preop)	Potassium (highest BL)
Bruising (preop)	WBC (preop)	Blood collection mode	CRP (preop)	WBC (preop)	Bicarbonate (lowest BL)
WBC (preop)	CRP (preop)	Bullae (preop)	creatinine (preop)	CRP (preop)	Urine output (BL)
CRP (preop)	creatinine (preop)	WBC (preop)	Haemoglobin (preop)	creatinine (preop)	creatinine (preop)
creatinine (preop)	Natrium (preop)	Blood products (baseline)	Age	Natrium (preop)	creatinine (BL)
Natrium (preop)	Haemoglobin (preop)	Center code	affected: head/neck	Haemoglobin (preop)	Noradrenaline (max BL)
Haemoglobin (preop)	BMI	Bullae (during 1st surgery)	Varicella	BMI	pH (lowest BL)
BMI	Center code	Haemoglobin (preop)	Size of skin defect (after first surgery)	Age	SBE (lowest BL)
Age	Age	CRP (preop)		affected: upper leg	Lactate (highest BL)
	affected: abdomen			Cardiovascular disease present	Crystalloids (BL)
	affected: upper leg				Accumulated fluids (BL)
	affected: lower leg				KDIGO stage
					creatinine (preadmission)
					WBC (preop)
**n = 11**	**n = 14**	**n = 11**	**n = 10**	**n = 12**	**n = 16**

Supplementary Table 4.

Prediction performance for clinical endpoints revolving around surgical aspects, patient management, and organ support. Ave. prec.: average precision, R^2 coefficient of determination, MAE: mean absolute error, MSE: mean squared error

	Risk of amputation	Size of skin defect (after first surgery)	Size of skin defect (maximal)	Days spent in ICU	Need for RRT (24h after ICU admission)
**Acc.**	0.503 (0.483, 0.546)	-	-	-	0.543 (0.493, 0.610)
**Prec.**	0.137 (0.000, 1.000)	-	-	-	0.467 (0.000, 1.000)
**Recall**	0.020 (0.000, 0.106)	-	-	-	0.109 (0.000, 0.250)
**F1-score**	0.033 (0.000, 0.182)	-	-	-	0.168 (0.000, 0.345)
**Brier**	0.113 (0.092, 0.136)	-	-	-	0.105 (0.083, 0.125)
**Ave. prec.**	0.261 (0.159, 0.411)	-	-	-	0.385 (0.238, 0.540)
**R^2**	-	0.120 (0.044, 0.178)	0.179 (0.100,0.234)	0.018 (-0.063,0.056)	-
**MAE**	-	3.500 (3.144, 3.831)	4.383 (3.905, 4.879)	7.047 (6.194,7.976)	-
**MSE**	-	24.086 (16.334, 34.611)	43.328 (27.789, 66.054)	111.952 (65.077, 172.308)	-
